# Emotional Well-Being and Glycemic Control in People with Diabetes After a Multidisciplinary Hybrid Education

**DOI:** 10.3390/healthcare14020198

**Published:** 2026-01-13

**Authors:** Carmen Amelia Ruiz-Trillo, Ana Pérez-Morales, Ana Cortés-Lerena, Pilar Santa Cruz-Álvarez, Mónica Enríquez-Macias, Manuel Pabón-Carrasco, Miguel Garrido-Bueno, Rocío Romero-Castillo, Virginia Bellido

**Affiliations:** 1 Unit of Endocrinology and Nutrition, Virgen del Rocío University Hospital, 41013 Seville, Spain; camelia.ruiz.sspa@juntadeandalucia.es (C.A.R.-T.); anam.perez.morales.sspa@juntadeandalucia.es (A.P.-M.); ana.cortes.sspa@juntadeandalucia.es (A.C.-L.); mp.santacruz.sspa@juntadeandalucia.es (P.S.C.-Á.); monica.enriquez.sspa@juntadeandalucia.es (M.E.-M.); virginiabellido@gmail.com (V.B.); 2 Research Group PAIDI-CTS-1050: “Complex Care, Chronicity and Health Outcomes”, Department of Nursing, Faculty of Nursing, Physiotherapy and Podiatry, University of Seville, 41004 Seville, Spain; rromeroc@us.es; 3 Instituto de Biomedicina de Sevilla (IBiS), Hospital Universitario Virgen del Rocío, CSIC, Universidad de Sevilla, 41004 Seville, Spain

**Keywords:** type 1 diabetes mellitus, patient education, remote consultation, nurses

## Abstract

**Highlights:**

**What are the main findings?**

- Multidisciplinary hybrid education with continuous glucose monitoring modestly improved HbA1c, especially in participants with baseline HbA1c > 8%, and older age plus higher initial HbA1c predicted a greater benefit.
- Diabetes-related quality of life, treatment satisfaction, and hypoglycemia awareness improved after the intervention.

**What are the implications of the main findings?**

- A multidisciplinary hybrid education model integrated with continuous glucose monitoring is a feasible and effective strategy to enhance metabolic control and psychosocial outcomes in adults with type 1 diabetes.
- These findings support implementing similar programs in routine care and justify future randomized studies to confirm and extend these results.

**Abstract:**

Background/Objectives: Multidisciplinary hybrid educational programs combined with continuous glucose monitoring may contribute to improved self-management in adults with type 1 diabetes mellitus (T1DM); however, real-world evidence remains limited. This study assessed the effects of an educational intervention integrated with continuous glucose monitoring on glycemic control and patient-reported outcomes in adults with T1DM. Methods: We conducted a single-group quasi-experimental study including 210 adults with T1DM from a public hospital. The nurse-led hybrid intervention consisted of a 2-h in-person group educational session followed by an individual telematic follow-up session. All participants used continuous glucose monitoring. The primary outcome was the change in HbA1c at 9 months. Secondary outcomes included continuous glucose monitoring metrics, diabetes-related quality of life, treatment satisfaction, and hypoglycemia awareness. Results: HbA1c showed a statistically significant but modest reduction from 7.70 ± 1.10% to 7.45 ± 0.91% following the intervention (*p* = 0.003). No statistically significant changes were observed in continuous glucose monitoring metrics, including time in range, time below and above range, mean glucose, glycemic variability, or sensor wear time. In terms of emotional well-being, treatment satisfaction increased significantly (8.17 ± 7.86 vs. 12.73 ± 5.49; *p* < 0.001), and the Clarke score showed a statistically significant but modest decrease (2.49 ± 1.90 vs. 2.12 ± 1.88; *p* = 0.017). Although the overall quality of life score did not change significantly, statistically significant differences were observed in several subscales, including satisfaction, impact, and diabetes-related concern. Conclusions: A multidisciplinary hybrid educational intervention integrated with continuous glucose monitoring was associated with modest improvements in HbA1c and statistically significant, though limited, enhancements in quality of life, treatment satisfaction, and hypoglycemia awareness in adults with T1DM. These findings suggest that similar educational models may have a supportive role in routine care.

## 1. Introduction

Diabetes mellitus is a chronic metabolic disorder characterized by elevated blood sugar levels due to the inability to produce or use insulin [1]. Diabetes not only represents a physical health problem but it also has profound behavioral, psychological, and social consequences, so living with this disease can significantly affect people’s emotional well-being and quality of life [2].

Managing diabetes is a complex and demanding challenge, as it requires specific knowledge and skills to address it effectively, solve problems, and minimize associated risks. Effective management of the disease often requires a multifaceted approach that includes not only blood glucose control but also psychological well-being, as the condition significantly affects daily life, requiring constant monitoring and lifestyle adjustments, which can be stressful and overwhelming [3].

Therapeutic education is an essential tool to include both patients and their families in the treatment of diabetes [4,5]. Structured education plays a critical role in introducing the use of advanced technologies applied in this field, such as the intermittent-scanning continuous glucose monitoring system (isCGM), which has revolutionized diabetes management by providing real-time glucose levels and a more comprehensive view of glucose trends and patterns, allowing for better-informed treatment decisions and better glycemic control [6].

The use of glucose monitoring with isCGM devices as an educational tool requires a structured program that trains patients in the use of these systems and the information they provide, thus optimizing the results obtained [6]. In randomized clinical trials, compared to capillary glycemic self-monitoring, a significant improvement in the number of hypoglycemic events and time in hypoglycemia was observed, although no change in glycated hemoglobin (HbA1c) levels was found. However, in actual clinical practice, several studies have documented a decrease in HbA1c levels [7].

Given this gap in knowledge about the effectiveness of multidisciplinary hybrid patient education in combination with isCGM, the aim of this study was to analyze the effects of multidisciplinary hybrid patient education with isCGM on glycemic control and patient-reported outcomes in people with diabetes type 1 mellitus (T1DM).

## 2. Materials and Methods

### 2.1. Study Design and Sampling

This was a single-center, uncontrolled, single-group, non-randomized and quasi-experimental study. It was conducted to analyze the influence of multidisciplinary hybrid patient education in people with diabetes.

Participants were recruited through consecutive sampling and eligibility criteria were applied. These included age (>18 years), duration of diabetes (>1 year, to avoid including individuals recently diagnosed), use of insulin therapy, attendance of at least one therapeutic education session on nutrition, and being new users of isCGM. This recruitment was carried out in Virgen del Rocío University Hospital. It is a public leading hospital where the laboratories follow the ISO 15189 regarding quality and technical competence [8], in southern Spain, in January 2023. Sample size calculations for this study were performed considering a 95% confidence level, a margin of error of 5%, and a variability of 50% for a more conservative scenario, so a sample size of 200 participants was established.

### 2.2. Characteristics of the Intervention

The intervention was conducted between January and May 2023 and consisted of a multidisciplinary therapeutic education program integrated into routine clinical care. The program followed a hybrid educational model and included an initial two-hour in-person group session followed by an individual telematic educational reinforcement session conducted 30 days later.

The initial face-to-face group session was held in a designated hospital education room and was primarily led by physicians specialized in diabetes care, with a multidisciplinary approach. This session focused on structured diabetes self-management education, including comprehensive training on the use and interpretation of intermittent-scanning continuous glucose monitoring systems, understanding glucose trends and variability, and practical strategies for the prevention and management of hypo- and hyperglycemia.

The subsequent individual telematic session was delivered online and constituted a nurse-led follow-up, aimed at reinforcing previously acquired knowledge, resolving individual doubts, reviewing glucose data, and supporting personalized self-management and treatment adjustments when necessary. To enhance adherence and facilitate self-care, all participants or their caregivers received written educational materials addressing the management of hypoglycemic and hyperglycemic events.

In addition, participants were granted access to the LibreView platform [9], which enabled remote monitoring of glucose data and eliminated the need for manual data downloads. All participants were provided with isCGM devices, model FreeStyle Libre 2 [10] and had uninterrupted access to the system throughout the entire follow-up period. The use of isCGM was part of standard clinical practice and was fully integrated into the educational intervention, allowing continuous assessment of glucose levels in real-life conditions throughout the study period.

The intervention was delivered by a multidisciplinary healthcare team from the Endocrinology and Nutrition Unit. The team comprised endocrinologists specialized in diabetes, who were responsible for patient selection, clinical oversight, and therapeutic decision-making; and specialized diabetes nurses, who led the structured educational sessions, conducted device training, performed data review through the remote monitoring platform, and provided individualized educational reinforcement and follow-up.

### 2.3. Data Collection

All data were collected using a combination of validated questionnaires and clinical measurements. Sociodemographic and clinical variables such as sex (man/woman), age (years), attendance to previous structured education programs (pre-education attendance) (yes/no), duration of diabetes (years), type of treatment (multiple doses of insulin/continuous subcutaneous infusion of insulin) were registered.

The primary outcome measured in this study was change in HbA1c levels (%) from baseline to the end of the study period. This was used to assess the effectiveness of educational intervention on glycemic control, along with the following data from the isCGM-derived metrics. Baseline values were calculated using data from the first 14 consecutive days of isCGM use prior to the educational intervention. Post-intervention CGM metrics were calculated using data from the final 14 consecutive days available at the end of the follow-up period (approximately 9 months). From these periods, the following ambulatory glucose profile metrics were extracted: time in range (TIR) (target ≥ 70%), time above range (TAR) (<25% for >180 mg/dL and <5% for >250 mg/dL), time below range (TBR) (<4% for <70 mg/dL and <1% for <54 mg/dL), glucose management indicator (GMI) (target around 7%), coefficient of variation (CV) (<36%), mean glucose levels, and sensor wear time (%).

Secondary outcome measures included (1) changes in quality of life, (2) changes in satisfaction with diabetes treatment, (3) changes in awareness of inadvertent hypoglycemia, (4) the proportion of participants who achieved a reduction in HbA1c levels, and (5) the presence of comorbidities.

(1) The Spanish Version of the Diabetes Quality of Life Questionnaire (EsDQOL) is a 43-element Likert-type instrument with seven options that is used to measure the quality of life of people with diabetes. It is the Spanish version of the DQOL questionnaire, in which reproducibility or reliability was evaluated using internal consistency (Cronbach’s alpha) and the test–retest using Pearson’s correlation coefficient. The overall internal consistency of the first version of the questionnaire was 0.90 [11].
(2) The Diabetes Treatment Satisfaction Questionnaire (DTSQ) is used to measure people’s satisfaction with their diabetes treatment, it consists of eight items rated on a Likert-type scale from 0 to 6. Cronbach’s alpha coefficients were 0.79 and 0.85, and the test–retest reliability was 0.64 and 0.68 for the state and change versions, respectively [12].
(3) The Clarke test is used to measure the number of episodes of non-conscious hypoglycemia using an 8-item questionnaire. Responses are classified as either normal (A) or abnormal (R), and the total score of R determines the person’s perception of hypoglycemia: 1 to 2R indicates normal perception, 3R indicates an indeterminate category, and 4R indicates abnormal perception. The internal consistency of the questionnaire, as measured by Cronbach’s alpha, was 0.75. In addition, the reliability of the test–retest showed a correlation coefficient of r = 0.81 and the correlations between the questionnaire score and the frequency of severe and non-severe hypoglycemic events were r = 0.47 and r = 0.77, respectively [13].

### 2.4. Data Analysis

The unit of assignment in this study is the individual, and no randomization method was used to assign units to the study conditions; therefore, all participants received the same educational intervention and follow-up. To minimize the potential bias induced by lack of randomization, a review and follow-up method was used [14]. Therefore, we conducted a continuous review of the data and monitored the intervention process to ensure consistency and quality in the implementation of the study. Since the unit of analysis does not differ from the allocation unit, it was not necessary to use additional analytical methods to adjust standard error estimates or employ multilevel analysis.

Due to the nature of the intervention, blinding of participants and staff was not possible. However, the data analysis was performed blindly using the Statistical Package for the Social Sciences (SPSS) software version 29.0.1.0 [15]. To ensure the robustness of the data analysis, the Kolmogorov–Smirnov normality test was applied to all quantitative variables because the sample size exceeded 50 participants [16]. This test revealed that the variables followed a normal distribution (*p* > 0.05), which motivated the use of appropriate parametric statistical methods for subsequent analyses.

Correlation analyses were performed to assess the associations between age and pre-post changes in glycemic control- and continuous glucose monitoring-derived outcomes using Pearson’s correlation coefficient. Sex-related differences in pre-post changes in glycemic control- and emotional well-being-related outcomes were evaluated using independent samples *t*-tests. For *t*-tests, mean differences, standard errors, 95% confidence intervals, and effect sizes were calculated, with effect sizes expressed as Cohen’s d. All statistical tests were two-tailed, and statistical significance was set at *p* < 0.05.

### 2.5. Study Considerations

This study was carried out in accordance with the ethical standards of the Declaration of Helsinki [17]. Acceptance of informed consent for participation in the research was also requested. This research was also carried out following the Transparent Reporting of Evaluations with Non-Randomized Designs (TREND) statement and checklist (Supplementary S1) [18]. It also obtained approval from the relevant ethics committee (ID: 0101-N-22).

## 3. Results

### 3.1. Characteristics of the Sample

In this study, 250 participants were initially contacted and selected for their eligibility. Of those, 210 were deemed eligible to participate, while 40 were not eligible according to the predefined criteria. There were no participants who declined to participate once they were deemed eligible, leading to the enrollment of all 210 eligible participants in the study. These participants were assigned to the same educational intervention condition, designed to improve their understanding and management of diabetes through rapid glucose monitoring education. There were no losses of participants in this study.

The diabetes-related sociodemographic and baseline characteristics of these participants were documented in detail prior to the start of the educational sessions (Table 1).

In addition, the presence of dyslipidemia (no n = 128, 61.0%; yes n = 82, 39.0%), diabetic retinopathy (no n = 153, 72.9%; yes n = 57, 27.1%), laser treatment (no n = 166, 79.0%; yes n = 44, 21.0%), neuropathy (no n = 189, 90.0%; yes n = 21, 10.0%), diabetic nephropathy (no n = 199, 94.8%; yes n = 11, 5.2%), coronary heart disease (no n = 205, 97,6%; yes n = 5, 2.4%), peripheral vascular disease (no n = 204, 97.1%; yes n = 6, 2.9%), cerebrovascular disease (no n = 207, 98.6%; yes n = 3, 1.4%), and diabetic foot (no n = 206, 98.1%; yes n = 4, 1.9%).

### 3.2. Effect of the Intervention in Glycemic Control

Changes in variables related to glycemic control were evaluated before and after the intervention (Table 2). HbA1c levels showed a statistically significant reduction, decreasing from 7.70 ± 1.10% at baseline to 7.45 ± 0.91% post-intervention (*p* = 0.003). In contrast, isCGM-derived metrics remained largely unchanged over the study period.

TIR showed similar values in the pre- and post-intervention assessments (63.77 ± 13.28% vs. 63.42 ± 15.15%, *p* = 0.086). TAR exhibited minor variations, with TAR 180–250 mg/dL showing a small change that approached statistical significance (22.52 ± 8.86% vs. 22.64 ± 9.51%, *p* = 0.054), while TAR > 250 mg/dL remained stable (8.53 ± 8.31% vs. 8.65 ± 9.84%, *p* = 0.103). Measures of hypoglycemia exposure also showed no significant differences, with comparable values for TBR 54–69 mg/dL (4.46 ± 3.84% pre-intervention vs. 4.54 ± 4.33% post-intervention, *p* = 0.483) and TBR <54 mg/dL (0.71 ± 1.22% vs. 0.65 ± 1.30%, *p* = 0.510).

Mean glucose, GMI, and CV values were similar at both time points. Mean glucose was 155.10 ± 24.77 mg/dL before the intervention and 155.70 ± 28.57 mg/dL after the intervention (*p* = 0.756), while GMI changed from 7.01 ± 0.59 to 7.04 ± 0.66 (*p* = 0.153), and CV from 37.81 ± 13.49% to 39.29 ± 20.67% (*p* = 0.452). Sensor wear time remained high and comparable between evaluations (90.66 ± 16.99% vs. 92.00 ± 13.19%, *p* = 0.971).

There were no statistically significant differences between men and women for changes in HbA1c (*p* = 0.233), TIR (*p* = 0.358), TAR (180–250 mg/dL) (*p* = 0.555), TAR (>250 mg/dL) (*p* = 0.698), TBR (54–69 mg/dL) (*p* = 0.435), TBR (<54 mg/dL) (*p* = 0.949), CV (*p* = 0.279), GMI (*p* = 0.329), mean glucose (*p* = 0.343), or sensor wear time (*p* = 0.145). Effect sizes were small across all comparisons, with Cohen’s d values ranging from −0.074 to 0.352 (Supplementary S2).

Correlation analyses between age and pre-post changes in glycemic control-related outcomes showed no significant associations between age and changes in HbA1c, TIR, TAR, TBR, CV, GMI, mean glucose, or sensor wear time. Significant correlations were observed among changes in CGM-derived metrics. Changes in TIR were negatively correlated with changes in TAR (180–250 mg/dL) and TAR (>250 mg/dL), and with changes in GMI and mean glucose. Changes in TAR were positively correlated with changes in GMI and mean glucose. Changes in TBR (54–69 mg/dL) and TBR (<54 mg/dL) were strongly correlated with each other and with changes in CV. Sensor wear time was positively correlated with changes in TIR and negatively correlated with changes in hypoglycemia metrics (Supplementary S3).

### 3.3. Influence of the Intervention in Emotional Well-Being

Changes in variables related to emotional well-being were assessed before and after the intervention (Table 3). Clarke score showed a statistically significant decrease, from 2.49 ± 1.90 in the pre-intervention period to 2.12 ± 1.88 post-intervention (*p* = 0.017). DTSQ scores increased significantly following the intervention, rising from 8.17 ± 7.86 to 12.73 ± 5.49 (*p* < 0.001).

No significant changes were observed in the overall EsDQOL score, which increased from 71.41 ± 54.76 at baseline to 95.82 ± 25.30 post-intervention (*p* = 0.759). However, significant differences were detected in several EsDQOL subscales. The satisfaction subscale showed a significant increase from 37.74 ± 9.60 to 51.28 ± 9.50 (*p* < 0.001), while the impact subscale decreased from 46.91 ± 10.77 to 43.94 ± 9.63 (*p* = 0.010). The diabetes concern subscale also decreased significantly, from 10.68 ± 3.53 to 9.94 ± 3.61 (*p* = 0.004). In contrast, no statistically significant changes were observed in the social anxiety subscale (13.77 ± 6.10 pre-intervention vs. 12.40 ± 5.78 post-intervention, *p* = 0.186).

There were no statistically significant differences between men and women for changes in Clarke Score (*p* = 0.474), DTSQ Score (*p* = 0.493), EsDQOL total score (*p* = 0.720), or its subscales of satisfaction (*p* = 0.221), impact (*p* = 0.989), diabetes concern (*p* = 0.246), and social anxiety (*p* = 0.780). Effect sizes were small across all comparisons (Cohen’s d ranging from −0.100 to 0.104) (Supplementary S4).

## 4. Discussion

In this study, the relevance of adopting a holistic and multidisciplinary perspective in the management of people with type 1 diabetes is acknowledged. The treatment of type 1 diabetes mellitus requires strict glycemic control as well as careful attention to patients’ psychological well-being [2]. This study suggests that multidisciplinary educational interventions combined with rapid glucose monitoring may contribute to modest improvements in both dimensions of diabetes care [5].

A statistically significant but modest reduction in HbA1c levels supports the potential effectiveness of multidisciplinary educational intervention with isCGM in improving glycemic control [5], a finding that is consistent with another study reporting that this approach was associated with improvements in glycemic control and self-management behaviors in people with diabetes [19]. Specifically, our study observed an average reduction of 0.3% in HbA1c among all participants, with a more pronounced reduction of 0.9% in those with baseline HbA1c levels greater than 8%. These results suggest that individuals with poorer initial glycemic control may experience comparatively greater benefit from such interventions. Similar observations were reported in another study, which identified several lifestyle factors associated with suboptimal glycemic control, including insufficient blood glucose monitoring [20].

Older participants and those with higher baseline HbA1c levels demonstrated comparatively better outcomes, indicating that targeted interventions may be particularly beneficial for these subgroups. This finding points to the potential value of personalized education programs tailored to specific demographic and clinical characteristics, which may modestly enhance the overall effectiveness of diabetes management strategies. This perspective is also supported by another study that emphasizes the importance of adopting personalized medicine approaches [21].

It is noteworthy that the statistically significant reduction observed in HbA1c was not accompanied by parallel significant changes in some isCGM-derived metrics, as reported in a previous study [22]. This apparent discrepancy between laboratory-based and sensor-based measures of glycemic control may reflect differences in the physiological constructs captured by each metric. HbA1c provides an integrated measure of glycemic exposure over several months, whereas CGM metrics reflect shorter-term glucose patterns and variability [23]. In addition, factors such as interindividual biological variability, red blood cell turnover, and measurement sensitivity may contribute to discordant findings [24].

Improvements in psychological well-being and quality of life are especially relevant, as managing type 1 diabetes mellitus represents not only a physical but also an emotional challenge. The study identified statistically significant improvements in quality of life, as assessed by the EsDQOL, as well as increased satisfaction with treatment, reflected in DTSQ scores. These findings reinforce the importance of addressing the emotional and psychological components of diabetes care, which can meaningfully influence health-related outcomes [2,11,12].

### 4.1. Limitations and Strengths

Our study presents several limitations, including the absence of a control group and the quasi-experimental design. These limitations warrant cautious interpretation of the results and indicate the need for further research to corroborate these observations [25].

Despite these limitations, the principal strength of the study lies in its comprehensive approach, combining multidisciplinary education with advanced glucose monitoring technology, thereby addressing both glycemic control and psychological well-being in people with type 1 diabetes [26]. The rigorous methodological design, supported by a robust sample size and the use of validated questionnaires, contributes to the reliability of the data. In addition, personalized and continuous education delivered through group sessions and telematic follow-ups represents another notable strength of this study.

### 4.2. Implications for Practice and Research

Future research should prioritize randomized controlled trials to further validate these findings and to examine the long-term benefits of such interventions. Additionally, it would be useful to explore how various demographic and clinical variables may influence the effectiveness of educational programs [27]. This line of inquiry could help refine and adapt interventions to maximize their impact across diverse populations [21].

Overall, this study suggests that the integration of multidisciplinary education with advanced glucose monitoring technologies may enable healthcare providers to deliver more effective and comprehensive care, potentially contributing to improved physical and emotional health outcomes for people with diabetes.

## 5. Conclusions

This study suggests that multidisciplinary therapeutic education programs, particularly those incorporating modern diabetes technologies such as instant glucose monitoring systems, may be associated with statistically significant but overall modest improvements in both glycemic control and psychological well-being in people with type 1 diabetes mellitus. These findings support the potential value of the broader implementation of such educational interventions to enhance the overall management and quality of life of individuals living with type 1 diabetes mellitus. The integration of technology-focused education with conventional diabetes care appears to represent a promising, though still evolving, approach to improving health-related outcomes and may merit consideration in clinical practice.

## Figures and Tables

**Table 1:** Baseline characteristics of the participants (n = 210).

Variable	Category	Proportion (n (%))
Sex	Man	90 (42.9)
	Woman	120 (57.1)
Age	Mean	44.5
	Standard deviation	12.4
Pre-education attendance	Yes	141 (67.3)
	No	69 (32.7)
Duration of diabetes	Mean	17.5
	Minimum–maximum	11–29
Type of treatment	Multiple doses of insulin	194 (92.4)
	Continuous subcutaneous infusion of insulin	16 (7.6)

**Table 2:** Changes in variables related to glycemic control.

Variable	Pre-Intervention (Mean ± SD)	Post-Intervention (Mean ± SD)	*p*-Value
HbA1c (%)	7.70 ± 1.10	7.45 ± 0.91	0.003
TIR (%)	63.77 ± 13.28	63.42 ± 15.15	0.086
TAR (180–250 mg/dL) (%)	22.52 ± 8.86	22.64 ± 9.51	0.054
TAR (>250 mg/dL) (%)	8.53 ± 8.31	8.65 ± 9.84	0.103
TBR (54–69 mg/dL) (%)	4.46 ± 3.84	4.54 ± 4.33	0.483
TBR (<54 mg/dL) (%)	0.71 ± 1.22	0.65 ± 1.30	0.510
Mean glucose (mg/dL)	155.10 ± 24.77	155.70 ± 28.57	0.756
GMI	7.01 ± 0.59	7.04 ± 0.66	0.153
CV (%)	37.81 ± 113.49	39.29 ± 290.67	0.452
Sensor wear time (%)	90.66 ± 16.99	92.00 ± 13.19	0.971

Note: *p*-value was calculated using paired *t*-tests. CV: Coefficient of Variation; GMI: Glucose Management Indicator; HbA1c: Glycated Hemoglobin; TAR: Time Above Range; TBR: Time Below Range; TIR: Time In Range.

**Table 3:** Changes in variables related to emotional well-being.

Variable	Pre-Intervention	Post-Intervention	*p*-Value
Clark Score	2.49 ± 1.90	2.12 ± 1.88	0.017
DSTQ Score	8.17 ± 7.86	12.73 ± 5.49	<0.001
EsDQOL Score	71.41 ± 54.76	95.82 ± 25.30	0.759
**EsDQOL Subscales:**			
Satisfaction	37.74 ± 9.60	31.28 ± 9.50	<0.001
Impact	46.91 ± 10.77	43.94 ± 9.63	0.010
Diabetes concern	10.68 ± 3.53	9.94 ± 3.61	0.004
Social anxiety	13.77 ± 6.10	12.40 ± 5.78	0.186

Note. *p*-value was calculated using paired *t*-tests. DTSQ: Diabetes Treatment Satisfaction Questionnaire; EsDQOL: Spanish Version of the Diabetes Quality of Life Questionnaire.

## Data Availability

Study data is available upon request to the corresponding authors.

## Supplementary Materials

The following supporting information can be downloaded at: https://www.mdpi.com/article/10.3390/healthcare14020198/s1, Supplementary S1: TREND Statement Checklist [18]; Supplementary S2: Changes in glycemic control-related outcomes by sex; Supplementary S3: Correlations between age and change in glycemic control-related outcomes; Supplementary S4: Changes in emotional well-being-related outcomes by sex.

## Abbreviations

The following abbreviations are used in this manuscript:

CI	Confidence Interval
CV	Coefficient of Variation
DQOL	Diabetes Quality of Life Questionnaire
DTSQ	Diabetes Treatment Satisfaction Questionnaire
EsDQOL	Spanish Version of the Diabetes Quality of Life Questionnaire
GMI	Glucose Management Indicator
HbA1c	Glycated hemoglobin
SPSS	Statistical Package for the Social Sciences
T1DM	Type 1 Diabetes Mellitus
TAR	Time Above Range
TBR	Time Below Range
TIR	Time In Range
TREND	Transparent Reporting of Evaluations with Non-Randomized Designs
