# Supplementary material for: Emotional Well-Being and Glycemic Control in People with Diabetes After a Multidisciplinary Hybrid Education
Source: Healthcare (Basel). 2026 Jan 13;14(2):198. doi: 10.3390/healthcare14020198 (PMC12841195; doi:10.3390/healthcare14020198)
Supplement: Supplementary file 1 [file healthcare-14-00198-s001.zip › Supplementary 2.pdf]

Supplementary S2

Changes in glycemic control-related outcomes by sex

	t	P value (two-tailed)	Mean difference	Standard error of the difference	Lower 95% CI	Upper 95% CI	Cohen's d
HbA1c	1.194	0.233	0.382	0.320	-0.247	1.012	0.119
TIR	0.923	0.358	6.088	6.595	-6.988	19.163	0.179
TAR (180-250 mg/dL)	0.593	0.555	1.385	2.335	-3.262	6.033	0.133
TAR (>250 mg/dL)	-0.389	0.698	-1.186	3.048	-7.227	4.855	-0.074
TBR1 (54–69 mg/dL)	0.785	0.435	0.858	1.094	-1.324	3.041	0.190
TBR2 (<54 mg/dL)	-0.064	0.949	-0.064	0.999	-2.118	1.990	-0.025
CV	1.091	0.279	3.178	2.913	-2.617	8.974	0.241
GMI	0.982	0.329	0.566	0.576	-0.582	1.714	0.228
Mean glucose	0.953	0.343	14.415	15.125	-15.578	44.409	0.187
Sensor wear time	1.473	0.145	11.907	8.083	-4.218	28.033	0.352

Note. P value was calculated using t-tests. CV: Coefficient of Variation; GMI: Glucose Management Indicator; HbA1c: Glycated Hemoglobin; T1DM: Type 1 Diabetes Mellitus; TAR: Time Above Range; TBR: Time Below Range; TIR: Time In Range; CI: Confidence Interval
