# Supplementary material for: Emotional Well-Being and Glycemic Control in People with Diabetes After a Multidisciplinary Hybrid Education
Source: Healthcare (Basel). 2026 Jan 13;14(2):198. doi: 10.3390/healthcare14020198 (PMC12841195; doi:10.3390/healthcare14020198)
Supplement: Supplementary file 1 [file healthcare-14-00198-s001.zip › Supplementary 3.pdf]

Supplementary S3

Correlations between age and change in glycemic control-related outcomes

		Age (years)	HbA1c	TIR	TAR (180-250 mg/dL)	TAR (>250 mg/dL)	TBR (54–69 mg/dL)	TBR (<54 mg/dL)	CV	GMI	Mean glucose	Sensor wear time
Age (years)	r	--										
	P value	.										
HbA1c	r	0.051	--									
	P value	0.312	.									
TIR	r	-0.045	0.097	--								
	P value	0.652	0.384	.								
TAR (180-250 mg/dL)	r	-0.176	0.178	-0.365**	--							
	P value	0.119	0.126	0.001	.							
TAR (>250 mg/dL)	r	-0.072	0.087	-0.175	0.490**	--						
	P value	0.462	0.420	0.071	<0.001	.						
TBR (54–69 mg/dL)	r	-0.072	-0.111	0.196	-0.220	-0.202	--					
	P value	0.558	0.378	0.112	0.073	0.094	.					
TBR (<54 mg/dL)	r	-0.117	-0.231	0.240	0.044	0.028	0.849**	--				
	P value	0.553	0.257	0.237	0.828	0.887	<0.001	.				
CV	r	0.046	0.061	-0.094	0.060	0.183	0.710**	0.720**	--			
	P value	0.683	0.596	0.408	0.598	0.096	<0.001	<0.001	.			
GMI	r	-0.027	0.345**	-0.410**	0.781**	0.570**	-0.231	-0.007	0.187	--		
	P value	0.813	0.003	<0.001	<0.001	<0.001	0.068	0.975	0.106	.		
Mean glucose	r	0.014	0.445**	0.265**	0.762**	0.548**	-0.332**	-0.044	0.156	0.916**	--	
	P value	0.890	<0.001	0.007	<0.001	<0.001	0.005	0.823	0.159	<0.001	.	
Sensor wear time	r	-0.040	-0.108	0.350**	0.130	0.048	0.450**	0.418*	0.271*	0.146	0.026	--
	P value	0.742	0.379	0.004	0.292	0.693	<0.001	0.037	0.023	0.251	0.828	.

Note. The correlation was calculated using Pearson's method. **: The correlation is significant at the 0.01 level (two-tailed); *: The correlation is significant at the 0.05 level (two-tailed). r: Pearson's coefficient; CV: Coefficient of Variation; GMI: Glucose Management Indicator; HbA1c: Glycated Hemoglobin; T1DM: Type 1 Diabetes Mellitus; TAR: Time Above Range; TBR: Time Below Range; TIR: Time In Range.
