# Supplementary material for: Emotional Well-Being and Glycemic Control in People with Diabetes After a Multidisciplinary Hybrid Education
Source: Healthcare (Basel). 2026 Jan 13;14(2):198. doi: 10.3390/healthcare14020198 (PMC12841195; doi:10.3390/healthcare14020198)
Supplement: Supplementary file 1 [file healthcare-14-00198-s001.zip › Supplementary 4.pdf]

Supplementary S4

Changes in emotional well-being-related outcomes by sex

	t	P value (two-tailed)	Mean difference	Standard error of the difference	Lower 95% CI	Upper 95% CI	Cohen's d
Clarke Score	-0.717	0.474	-0.115	0.161	-0.432	0.201	-0.060
DTSQ Score	-0.686	0.493	-0.517	0.753	-1.996	0.963	-0.055
EsDQOL Score	0.358	0.720	1.597	4.458	-7.160	10.353	0.031
EsDQOL Subscales:							
Satisfaction	1.225	0.221	1.966	1.605	-1.186	5.119	0.104
Impact	0.014	0.989	0.029	2.021	-3.942	4.000	0.001
Diabetes concern	-1.162	0.246	-0.594	0.511	-1.598	0.410	-0.100
Social anxiety	0.279	0.780	0.213	0.763	-1.285	1.711	0.024

Note. P value was calculated using t-tests. DTSQ: Diabetes Treatment Satisfaction Questionnaire; EsDQOL: Spanish Version of the Diabetes Quality of Life Questionnaire; CI: Confidence Interval
